# Surface Properties of Nanostructured, Porous ZnO Thin Films Prepared by Direct Current Reactive Magnetron Sputtering

**DOI:** 10.3390/ma11010131

**Published:** 2018-01-14

**Authors:** Monika Kwoka, Barbara Lyson-Sypien, Anna Kulis, Monika Maslyk, Michal Adam Borysiewicz, Eliana Kaminska, Jacek Szuber

**Affiliations:** 1 Institute of Electronics, Silesian University of Technology, 44-100 Gliwice, Poland; Barbara.Lyson-Sypien@polsl.pl (B.L.-S.); Anna.Kulis@polsl.pl (A.K.); Jacek.Szuber@polsl.pl (J.S.); 2 Institute of Electron Technology, 02-668 Warsaw, Poland; mmaslyk@ite.waw.pl (M.M.); mbory@ite.waw.pl (M.A.B.); eliana@ite.waw.pl (E.K.)

**Keywords:** ZnO nanostructures, reactive magnetron sputtering, XPS, surface chemistry

## Abstract

In this paper, the results of detailed X-ray photoelectron spectroscopy (XPS) studies combined with atomic force microscopy (AFM) investigation concerning the local surface chemistry and morphology of nanostructured ZnO thin films are presented. They have been deposited by direct current (DC) reactive magnetron sputtering under variable absolute Ar/O_2_ flows (in sccm): 3:0.3; 8:0.8; 10:1; 15:1.5; 20:2, and 30:3, respectively. The XPS studies allowed us to obtain the information on: (1) the relative concentrations of main elements related to their surface nonstoichiometry; (2) the existence of undesired C surface contaminations; and (3) the various forms of surface bondings. It was found that only for the nanostructured ZnO thin films, deposited under extremely different conditions, i.e., for Ar/O_2_ flow ratio equal to 3:0.3 and 30:3 (in sccm), respectively, an evident and the most pronounced difference had been observed. The same was for the case of AFM experiments. What is crucial, our experiments allowed us to find the correlation mainly between the lowest level of C contaminations and the local surface morphology of nanostructured ZnO thin films obtained at the highest Ar/O_2_ ratio (30:3), for which the densely packaged (agglomerated) nanograins were observed, yielding a smaller surface area for undesired C adsorption. The obtained information can help in understanding the reason of still rather poor gas sensor characteristics of ZnO based nanostructures including the undesired ageing effect, being of a serious barrier for their potential application in the development of novel gas sensor devices.

## 1. Introduction

Zinc oxide (ZnO) is one of the most popular transparent conductive oxides (TCO) having unique optical and electronic properties (wide energy band gap of 3.37 eV and large exciton binding energy of −60 meV). Moreover, among the TCO materials, ZnO exhibits high charge carrier mobility (from several to hundreds cm^2^/Vs). However, its precise value strongly depends on its forms and dimensionalities directly related to the preparation and deposition methods, what was nicely reviewed by Jagadish and Pearton [1].

The unique optical and electronic properties make zinc oxide a promising candidate mainly for selected optoelectronic devices [1] and solar cells [2]. In turn, having the electrical conductivity being sensitive to the composition of surrounding atmosphere, due to the adsorption/desorption processes on its surface, ZnO is a promising material for gas sensor application [3]. 

It is commonly known that the gas sensing performance of ZnO thin films strongly depends on their morphology, structure, and related surface nonstoichiometry. It is generally accepted that the high gas sensitivity can be achieved for the gas sensing material characterized by the internal structure exhibiting the largest surface-to-volume ratio. This condition can be fulfilled by using low dimensional nanostructures, especially 1D systems like nanowires, nanobelts, nanotubes, etc. [4,5]. In the literature, one can find numerous nanostructures reported for ZnO being fabricated using a wide array of techniques [6]. However, since the technology of 1D nanostructures elaboration is a time-consuming as well as relatively expensive task, their implementation is up to now rather unattractive for large-scale gas sensors device fabrication.

This is why special attention has recently been paid to other forms of ZnO with a large surface-to-volume ratio. From this point of view, ZnO porous thin films are well promising. 

Apart from sol-gel [7,8,9], sol-gel combined with spin coating [10,11], sol-gel combined with dip coating [12,13], and low temperature electrochemical deposition [14,15], direct current (DC) magnetron sputtering can be successfully used to obtain nanoporous ZnO thin films [16,17,18,19] having, among others, improved gas sensor characteristics [20,21], as was reviewed by Kumar et al. [22]. 

Using magnetron sputtering technology, the various porous TiO_2_ nanostructures, among other nanocolumnar and scaffold types, have also been recently obtained, however, mainly for their photovoltaics potential application [23,24], for which the expectations concerning the morphology and structure are rather different with respect to the gas sensors application, this being a main motivation of our studies.

Our recent Energy Dispersive X-ray (EDX) analysis of sputtered Zn/ZnO nanostructures fabricated at a constant total gas pressure, set argon-to-oxygen flow ratio, and changing gas flow values [19] confirmed that with increasing the gas flow, the respective oxygen content inside the obtained films also increased, probably as a result of the surface oxidation of the smaller grains. However, still, the relative O/Zn concentration was in the range of 0.42–0.80, meaning that even the final ZnO nanostructured porous thin films were rather very far from stoichiometry. 

Independently, it was also confirmed by various groups [19,20,24] that with increasing gas flows during deposition, as well as when argon partial pressure in the plasma increased, the grain size of ZnO nanostructures decreased. This phenomenon was also observed in our recent studies [19], as, for the highest Ar/O_2_ flow ratio (30:3), due to the increasing density of oxygen and related nucleation centers for new grains formation, dense nanoporous ZnO films were created. 

This last fact is absolutely crucial from the point of the potential applications of porous ZnO nanostructured thin films as gas sensing materials, since the strongest gas sensing effect mainly appears just at the subsurface of the sensing layer at the depth related to the Debye length of a few nm [4,5]. 

However, this information is not enough when trying to understand the still poorly known characteristics of ZnO nanostructures [23,24]. This is related to the fact that the gas sensing mechanism involves surface chemisorption, i.e., charges exchange between adsorbed gaseous species and the surface of porous ZnO nanostructures. This is why surface analytical methods able to give the information about the surface chemistry (including undesired contaminations) of ZnO nanostructured forms are required. 

Having all the above in mind, we have decided to perform comparative studies of the surface properties of the porous ZnO nanostructured thin films with various morphologies. In order to remove from the analysis the considerations related to different growth techniques, instead of collecting different ZnO morphologies grown by different methods, we chose to use different samples deposited by DC reactive magnetron sputtering, as developed by our group [19]. The application of specific deposition conditions, i.e., with a constant total gas pressure and a set argon-to-oxygen flow ratio of 10:1, while changing the Ar/O_2_ gas flow in the range from 3:0.3 to 30:3 (in sccm), enabled us to achieve various nanostructured hierarchical morphologies, varying by the degree of surface development. The growth mechanism relies on a high plasma concentration to achieve low kinetic energy of the atoms ejected from the target surface, resulting in a low adatom mobility, with which the self-shadowing effects yield nanoporous morphologies, as was already discussed in detail in our recent paper [19]. 

In order to examine reliably the surface properties of the porous ZnO nanostructured thin films, in this study, we have decided to use the surface sensitive methods i.e., X-ray photoelectron spectroscopy (XPS) combined with atomic force microscopy (AFM), having the information depth related to the Debye length [4,5]. The proposed approach is absolutely crucial for deeper insight to the local surface properties of ZnO nanostructures with a special emphasis on surface chemistry (including undesired surface contaminations) directly related to their surface morphology. 

Since the gas sensing effect involves surface chemisorption, a detailed characterization of the fundamental physico-chemical properties is required for the adequate design and construction of novel type gas sensor devices based on this material. The sensor effect appears just at the surface of the sensing material at the depth related to the Debye length [4,5], which is quite similar to the information depth for the XPS method.

## 2. Results and Discussion

**Figure 1:**
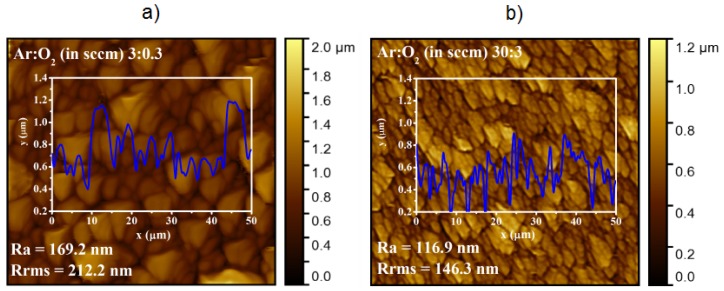
AFM images of nanostructured ZnO thin films deposited at the Ar/O_2_ gas flow of 3:0.3 (**a**) and 30:3 (**b**), respectively (in sccm); *R_a_* denotes arithmetical mean deviation of the assessed profile, whereas *R_rms_* is a root mean square roughness parameter.

At the beginning, the AFM images were recorded for the nanostructured ZnO thin films deposited at the different conditions, i.e., for Ar/O_2_ flow ratio ranging from 3:0.3 to 30:3 (in sccm). However, because the evident and most pronounced difference in AFM images has only been observed for the ZnO samples deposited at the extremely different conditions, i.e., for Ar/O_2_ flow ratio equal to 3:0.3 and 30:3 (in sccm), respectively, the detailed analysis of surface morphology has been proposed for these two selected nanostructured ZnO thin films, on the base of respective AFM images shown in Figure 1. 

The visual information shown in Figure 1 has been deeply confirmed on the base of selected AFM analytical parameters, i.e., the commonly used root mean square roughness parameter, *Rrms*, as well as the arithmetical mean deviation of the assessed profile, *R_a_*, defined as:

$$R_{a}=\frac{1}{L}\int_{0}^{L}\left|Z(x)\right|dx$$

where *Z*(*x*) represents the function that describes the depth profile, whereas *L* denotes length taken into account.

From the respective AFM images shown in Figure 1, it is evident that for the highest Ar/O_2_ flow ratio, the most dense ZnO nanostructures of highest porosity were obtained containing the well-recognized grains with a dimension of 100 nm, having a shape similar to nanoflowers, which in turn consist of densely packaged (agglomerated) nanograins having the average size in the range of 20–40 nm. It was related to the fact that only for the highest Ar/O_2_ flow does the highest amount of oxygen inhibit the growth and coalescence of Zn crystallites by promoting new nucleation centers at the growth front in the presence of oxygen adatoms. This leads to a decrease in the crystallite size, directly causing the modification of samples morphology, as explained in details in [19] based on XRD, TEM, and SEM measurements. In turn, the XPS experiments, being the main point of this research, have been performed in order to verify the local surface chemistry of nanostructured ZnO thin films. 

**Figure 2:**
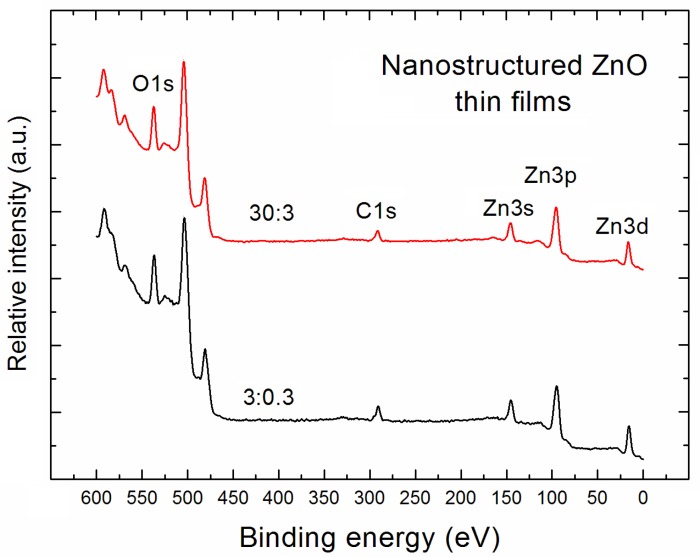
XPS survey spectra of nanostructured ZnO thin films deposited at the Ar/O_2_ gas flow of 3:0.3 and 30:3 (in sccm), respectively.

Within the XPS experiments, the survey spectra in the commonly used binding energy range (1200 eV) for the nanostructured ZnO thin films deposited at the different abovementioned Ar/O_2_ flow ratios, have been recorded. In general, the respective XPS survey spectra were very similar, apart from the nanostructured ZnO thin films deposited under extremely different conditions i.e., for Ar/O_2_ flow ratio equal to 3:0.3 and 30:3 (in sccm), respectively, for which an evident and the most pronounced difference has been observed, as for the case of AFM experiments. However, because the very large undesired background was observed—especially in the binding energy range 1200–600 eV, together with the contribution from undesired Auger electron emission line at 970 eV coming from the number of Auger transition O KLL—for the determination of relative concentration of main elements at the surface (in subsurface layers) of the nanostructured ZnO thin films, the survey spectra in the limited lower binding energy range (600 eV) were only taken into account. This is why Figure 2 only shows the XPS survey spectra in the limited lower binding energy range (600 eV) recalibrated with respect to XPS O1s spectral line obtained for our nanostructured ZnO thin films deposited under the abovementioned extremely different conditions.

The XPS survey spectra shown in Figure 2 mainly contain the contribution from XPS core level lines: O1s, Zn3s, Zn3p, and Zn3d, corresponding to the main elements of our nanostructured ZnO thin films. Moreover, what is crucial for our research, an evident undesired contribution of C1s XPS lines is observed, which confirmed the strong C contamination at the surface of nanostructured ZnO samples under investigation. However, apart from the above specific XPS lines in our spectra shown in Figure 2, one can also observe the additional peaks related to the Auger electron emission lines at ~570 eV, ~500 eV, and ~470 eV, corresponding to the Zn L_3_M_23_M_45_, Zn L_3_M_45_M_45_, and Zn L_2_M_45_M_45_ Auger electron transitions, respectively. 

As was mentioned above, using the XPS survey spectra shown in Figure 2, the relative concentration of main specific elements like O, Zn, and C, with respect to all the recognized surface atoms, in subsurface layers of our nanostructured ZnO thin films was determined using the following well-known analytical formulas [25,26]:

$$\frac{\left[O\right]}{\left[Zn]+[O]+[C\right]}=\frac{\frac{I_{O}}{ASF_{O}}}{\frac{I_{Zn}}{ASF_{Zn}}+\frac{I_{O}}{ASF_{O}}+\frac{I_{C}}{ASF_{C}}}\;\mathrm{and}\;\frac{\left[C\right]}{\left[Zn]+[O]+[C\right]}=\frac{\frac{I_{C}}{ASF_{C}}}{\frac{I_{Zn}}{ASF_{Zn}}+\frac{I_{O}}{ASF_{O}}+\frac{I_{C}}{ASF_{C}}}$$

and using the abovementioned intensity (height) *I_i_* of the O1s, C1s, and Zn3p core level lines (peaks), then corrected by the transmission function T(E) of CHA PHOIBOS 100 of 1.00, 0.90, and 0.85, respectively, and finally after taking into account the atomic sensitivity factors *ASF* related to the height of peaks for O1s (O.66), C1s (0.25), and Zn3p (0.4), respectively. The obtained data are summarized in Table 1. 

Of course, the above relative concentrations of the basic specific elements can be also be expressed as their total relative concentration (in %) in the subsurface region. On the basis of the information summarized in Table 1, one can notice that the relative concentration of O atoms with respect to all other surface atoms for our nanostructured ZnO thin films is rather similar because it only varies in the range of 0.25–0.29, being higher for the ZnO sample deposited at the highest gas flow ratio (30:3). 

In turn, the relative concentration of Zn atoms with respect to all the surface atoms for our nanostructured ZnO thin films is rather similar because only varies in the range of 0.48–0.55, being higher for the nanostructured ZnO thin films deposited at highest gas flow ratio (30:3). Crucially, the respected difference in Zn concentration is more than two times larger with respect to the accuracy. 

In contrary to the above, the relative concentration of C atoms with respect to all the surface atoms for our nanostructured ZnO thin films is evidently different varies in the range 0.27–0.18, being evidently lower for the nanostructured ZnO thin films deposited at highest gas flow ratio (30:3). It means that the respected difference in C concentration is three times larger with respect to the accuracy. This last information is crucial because it helps to recognize and then interpret the role of undesired C contaminations at the surface of our nanostructured ZnO thin films. 

In general, the results described above prove that an evident nonstoichiometry appears in the surface/subsurface region of our nanostructured ZnO thin films, combined with the relatively high concentration of undesired C surface contaminations. This can be probably related to the existence of the specific additional forms of oxygen as well as carbon surface bondings.

**Figure 3:**
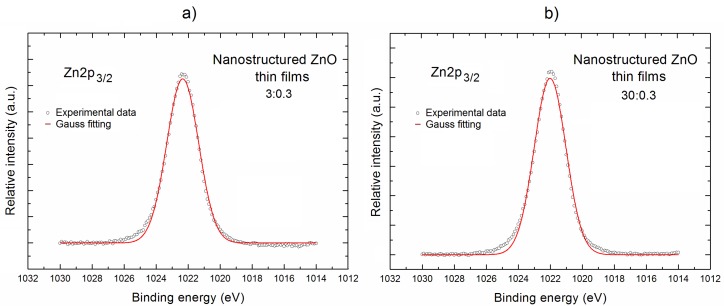
The XPS Zn2p_3/2_ lines after deconvolution using Gauss fitting for the nanostructured ZnO thin films deposited at the Ar/O_2_ gas flow of 3:0.3 (**a**) and 30:3 (**b**) (in sccm), respectively, having the most different Zn surface concentration.

**Figure 4:**
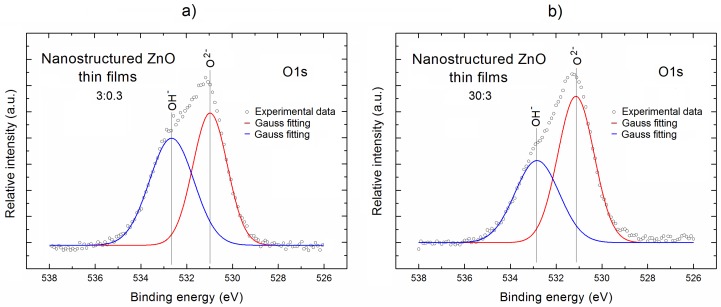
The XPS O1s lines after deconvolution using Gauss fitting for the two selected nanostructured ZnO thin films deposited at Ar/O_2_ gas flow of 3:0.3 (**a**) and 30:3 (**b**), respectively, having the most different total relative O concentration.

**Figure 5:**
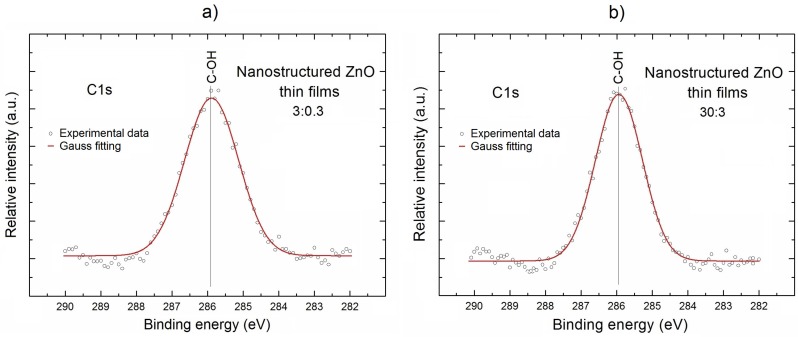
The XPS C1s lines after deconvolution using Gauss fitting for the selected nanostructured ZnO thin films deposited at Ar/O_2_ gas flow of 3:0.3 (**a**) and 30:3 (**b**), respectively, having the most different C total relative concentration.

In order to solve this issue, during the next step of our XPS research, we have focused on the more precise analysis of the local surface chemistry of our nanostructured ZnO thin films, with a special emphasis on the specific surface bonding. This analysis is based on the recorded XPS spectral lines: Zn2p, O1s, and C1s after their deconvolution. The deconvolution procedure was performed using the Casa XPS SPECS software. The obtained results are presented in Figure 3, Figure 4 and Figure 5, respectively, and interpreted below. 

Figure 3 shows the Zn2p_3/2_ XPS lines for our both nanostructured ZnO thin films (having the highest intensity among all the specific XPS Zn lines).

It is evident that shape of XPS Zn2p_3/2_ lines for our both samples look symmetrical. However, in order to verify the existence of any specific surface bonding, the decomposition procedure was performed for the XPS Zn2p_3/2_ lines (after the respective linear background subtraction) using Gauss fitting, and the obtained results are shown as the red curves in Figure 3. 

The deconvoluted XPS Zn2p_3/2_ lines for our both samples are characterized by a very large line fitting parameter (RMS = 0.995) being close to 1, having similar line widths for both ZnO samples at the level of ~2.4 eV, which confirms that only one component is observed always at the binding energy ~1022 eV, which corresponds to the zinc atoms of ZnO lattice at the surface. 

In turn, Figure 4 shows the O1s XPS lines for our both nanostructured ZnO thin films. It is evident that in contrary to XPS Zn2p_3/2_ lines, the XPS O1s lines exhibit an evident asymmetry, which is probably related to the existence of different forms of oxygen bondings at their surface. The detailed verification of potential forms of O bondings at the surface of our nanostructured ZnO thin films was performed on the basis of deconvolution of XPS O1s (after the respective linear background subtraction) using the Gauss fitting procedure, and the obtained results are shown as the red curves in Figure 4. 

For both nanostructured ZnO samples, the XPS O1s lines consist of two components, which are shown as the blue and red curves, respectively. The first one is located at the binding energy of ~531.0 eV and can be attributed to the O^2^^−^ ions in ZnO lattice, whereas the second one at binding energy ~532.5 eV corresponds to the oxygen atoms in OH^−^ groups at ZnO surface. The XPS line widths of recognized components for our both ZnO samples very similar and equal to 2.35 eV for the left component and 1.88 eV for the right component, respectively. 

Similar results concerning the components of O1s XPS line were obtained by Gazia et al. [27] for the spongelike nanostructured ZnO films deposited from the sputtered nanostructured zinc films. Using the components of XPS O1s lines shown in Figure 4, the relative area under them corresponding to the O^2^^−^ ions and OH^−^ groups was determined. For the nanostructured ZnO thin films deposited at lower Ar/O_2_ ratio (3:0.3), the contributions of OH^−^ groups and O^2^^−^ ions are almost comparable (~1.0). In turn, for the nanostructured ZnO thin film deposited at the highest Ar/O_2_ ratio (30:3), the relative concentration of O^2^^−^ ions over OH^−^ groups increases reaching the value ~1.5.

In should be underlined at this moment that the abovementioned information related to the existence of OH^−^ groups at the surface of our both ZnO samples remains in agreement with the information obtained from the XPS C1s lines for our both nanostructured ZnO thin films, which are shown in Figure 5. These XPS C1s lines confirm the existence of undesired C contaminations appearing at the surface of our nanostructured ZnO thin films after their exposition to the air atmosphere during the transportation between the deposition chamber and XPS chamber. 

It is evident that, in contrary to XPS Zn2p_3/2_ lines, but similar to the XPS O1s lines, the XPS C1s lines look symmetrical. However, in order to verify the existence of any specific surface bonding, their decomposition was performed (after the respective linear background subtraction) using the Gauss fitting procedure, and the obtained results are shown as the red curves in Figure 5. 

It is obvious that, for our both ZnO samples, after deconvolution of XPS C1s lines (with rather high line fitting parameter (RMS ~ 0.98)), only one component is observed at the binding energy of ~286 eV, having similar line widths of 1.84 eV for both ZnO samples, which can be attributed to the C–OH surface bondings [28]. 

This last information confirms that, in the case of our nanostructured ZnO thin films, one can observe the contribution from the two types of different hydroxyl groups (OH) at their surface, i.e., OH^−^ observed in XPS O1s peaks at binding energy ~532.5 eV, and C–OH groups observed in XPS C1s peaks at the binding energy of ~286 eV. The presence of these hydroxyl groups can lead to the variation of local surface chemistry of our nanostructured ZnO thin films.

The different C concentrations at the surface of our nanostructured ZnO thin films are related to their nonstoichiometry, which can be directly correlated with their local surface morphology. As was mentioned above, in the case of the nanostructured ZnO thin films deposited at the highest Ar/O_2_ ratio (30:3), having slightly higher nonstoichiometry (0.29/0.53), the highest porosity is observed, as the results of existence of densely packaged (agglomerated) nanograins having the average size in the range 20–40 nm, as shown in Figure 1. This is probably why in this case, the lowest (0.18) total relative C concentration at the surface was observed, what is related to the smallest surface area for carbon adsorption directly corresponding to the contribution of OH^−^ groups, because this ZnO sample adsorbs easier the OH^−^ groups at the surface.

This last observation was in opposite to the nanostructured ZnO thin films deposited at the lowest Ar/O_2_ ratio (3:0.3), containing the well-recognized grains with a dimension of 100 nm, for which the highest relative C concentration (0.27) was observed, even having only slightly lower relative O concentration (0.25). 

From the point of view of the possible gas sensing application of nanostructured ZnO thin films at this stage, one can conclude that ZnO nanostructures obtained at the highest Ar/O_2_ ratio (30:3), having the lowest level of C contaminations can be promising candidate for the detection of mainly oxidizing gases, especially in the presence of water vapor (H_2_O), because their nonstoichiometry corresponds to the higher concentration of oxygen vacancies, which always play a crucial role as the specific adsorption sites for various active oxidizing gases during the gas sensing process. It causes that these nanostructured ZnO thin films can be very sensitive mainly to the toxic gas species containing oxygen from the environment, like nitrogen dioxide (NO_2_).

This is crucial because the high undesired concentration of C contamination including C–OH species always play a role of undesired barrier for instance toxic gas adsorption, especially at the lower working temperature, and can additionally strongly affect the uncontrolled sensor ageing effect.

## 3. Materials and Methods

ZnO nanostructures were obtained by DC reactive magnetron sputtering using zinc target at 80 W DC power under 4 mbar total pressure and Ar/O_2_ gas flow at a set ratio (in sccm): 3:0.3; 8:0.8; 10:1; 15:1.5; 20:2; and 30:3. A 5’ long presputtering was performed before every deposition process to clean and stabilize the target surface. The thin films were prepared using a Surrey NanoSystems γ 1000 C reactor. The thicknesses of the films were in the range of 886 nm for the “3:0.3 sccm” sample (thickest film) to 570 nm for the “30:3 sccm” sample (thinnest film). With a constant deposition time of 1 h, this yielded deposition rates from 2.46 to 1.58 Å/s, respectively. The cathode voltages changed by 5%, from 400 V for 3:0.3 to 380 V for 30:3. The gas flows were controlled by dedicated Mass Flow Controllers and the system that utilized a capacitative Baratron manometer in a feed-back loop with a throttle valve (VAT, Haag, Switzerland) for pressure control. The deposition chamber was pumped by a cryogenic pump and the throttle valve was at the entrance to the pump. Such a setup enabled to obtain 10^−5^ Pa base vacuum and an independent control of the total gas pressure and the gas flow values. Si (100) wafers were used as the substrates. In order to remove any native oxides, prior to the ZnO nanostructured thin films deposition, the substrates were firstly degreased by boiling in selected organic solutions and bathed in the buffered HF solution to strip the oxide. The technological details concerning preparation of nanostructured ZnO thin films can be found elsewhere [19].

The surface chemistry together with the possible contaminations of the abovementioned nanostructured ZnO thin films have been examined by XPS method. In these studies, the commercial XPS spectrometer (SPECS, Berlin, Germany) equipped with the X-ray lamp (AlK_α_, 1486.6 eV, XR-50 model) and a concentric hemispherical analyzer (PHOIBOS-100 Model) was applied, pumped by oil-free pumping unit containing the Varian 110 model Scroll pump, Varian 551 model Turbo-pump, and Varian 300 model ion pump. The basic working pressure was below ~10^−^^8^ hPa, controlled with the Granville Phillips 360 model gas pressure system

The binding energies (BE) of all the registered XPS spectra have been calibrated to Au4f peak at 84.5 eV. Other experimental details have been described elsewhere [29,30,31]. 

The nanostructured ZnO thin films’ morphology was studied using AFM Bruker MultiMode 8 system (Bruker, Santa Barbara, CA, USA). It consists of MultiMode8 head completed with three scanners: AS-130VLR-2, AS-2VLR-2, and AS-05-2, having different scanning ranges (areas) and working with the NANOSCOPE V controller using the advanced original NanoScope V9.10 software. The MultiMode8 head is placed on a specific table (VT-102-2 model) equipped with pneumatic isolation system against vibrations, combined with the air compressor, which allows for the elimination of undesired mechanical vibrations of the surroundings.

## 4. Conclusions

In this paper, the information on the local surface chemistry of ZnO thin films, deposited by DC reactive magnetron sputtering under different Ar/O_2_ gas flow ratio, was obtained using the XPS method. Basing on these experimental results, we were able to obtain the crucial information on: (1) the total relative concentrations of main elements combined with nonstoichiometry; (2) the existence of undesired C surface contaminations; and (3) the various forms of surface bondings. What is extremely important is that the lowest amount of undesired C contamination was observed at the surface of our nanostructured ZnO thin films deposited at the highest Ar/O_2_ ratio, which can be directly correlated with their local surface morphology observed by SEM and related to the densely packaged (agglomerated) nanograins, yielding a smaller surface area for carbon absorption.

The information obtained in our studies can be very helpful in the interpretation of still rather poorly known gas sensor characteristics (mainly dynamic) of ZnO, which exhibits high electronic mobility (up to 2 cm^2^/V·s) and thus can be a very prospective gas sensor material, especially in the form of nanostructures. However, an exact gas sensor mechanism, including ageing effect in the case of various ZnO nanoforms, still remains unclear and requires further study.

## Figures and Tables

**Table 1:** The relative concentrations of all the main specific elements of nanostructured ZnO thin films in the subsurface layers.

Ar/O_2_ Ratio at Deposition of ZnO Thin Films (in sccm)	Relative Concentration of the Main Specific Elements		
	[O]/([Zn] + [O] + [C])	[Zn]/([Zn] + [O] + [C])	[C]/([Zn] + [O] + [C])
3:0.3	0.25 ÷ 0.03	0.48 ÷ 0.03	0.27 ÷ 0.03
30:3	0.29 ÷ 0.03	0.53 ÷ 0.03	0.18 ÷ 0.03
